# Supplementary material for: A stargate mechanism of Microviridae genome delivery unveiled by cryogenic electron tomography
Source: bioRxiv. 2024 Jun 11:2024.06.11.598214. Preprint. [Version 1] doi: 10.1101/2024.06.11.598214 (PMC11195240; doi:10.1101/2024.06.11.598214)
Supplement: Supplement 1 [file media-1.pdf]

A stargate mechanism of *Microviridae* genome delivery unveiled by cryogenic electron tomography

Pavol Bardy *et al.*

Supplementary information

Description of Supp Data
Supp Figures S1-S8
Supp Tables S1-S4
Supplementary References

Description of Supp Data

Supp Movie S1: Tomogram of Ebor particles attached to outer membrane vesicles formed from disrupted cells of *Rhodobacter capsulatus*, strain B10.

Supp Movie S2: Tomogram of Ebor particles attached to the host cell of *Rhodobacter capsulatus*, strain B10.

Supp Movie S3: Depiction of the stargate opening of Ebor particle, side view.

Supp Movie S4: Depiction of the stargate opening of Ebor particle, top view.

Supp Data S1: Mass spectrometry analysis of purified virions of Ebor.

Supp Data S2: Database of *Microviridae* genomes used for the phylogeny analysis.

Supp Data S3: A model of the trimeric protrusion predicted by AlphaFold2.

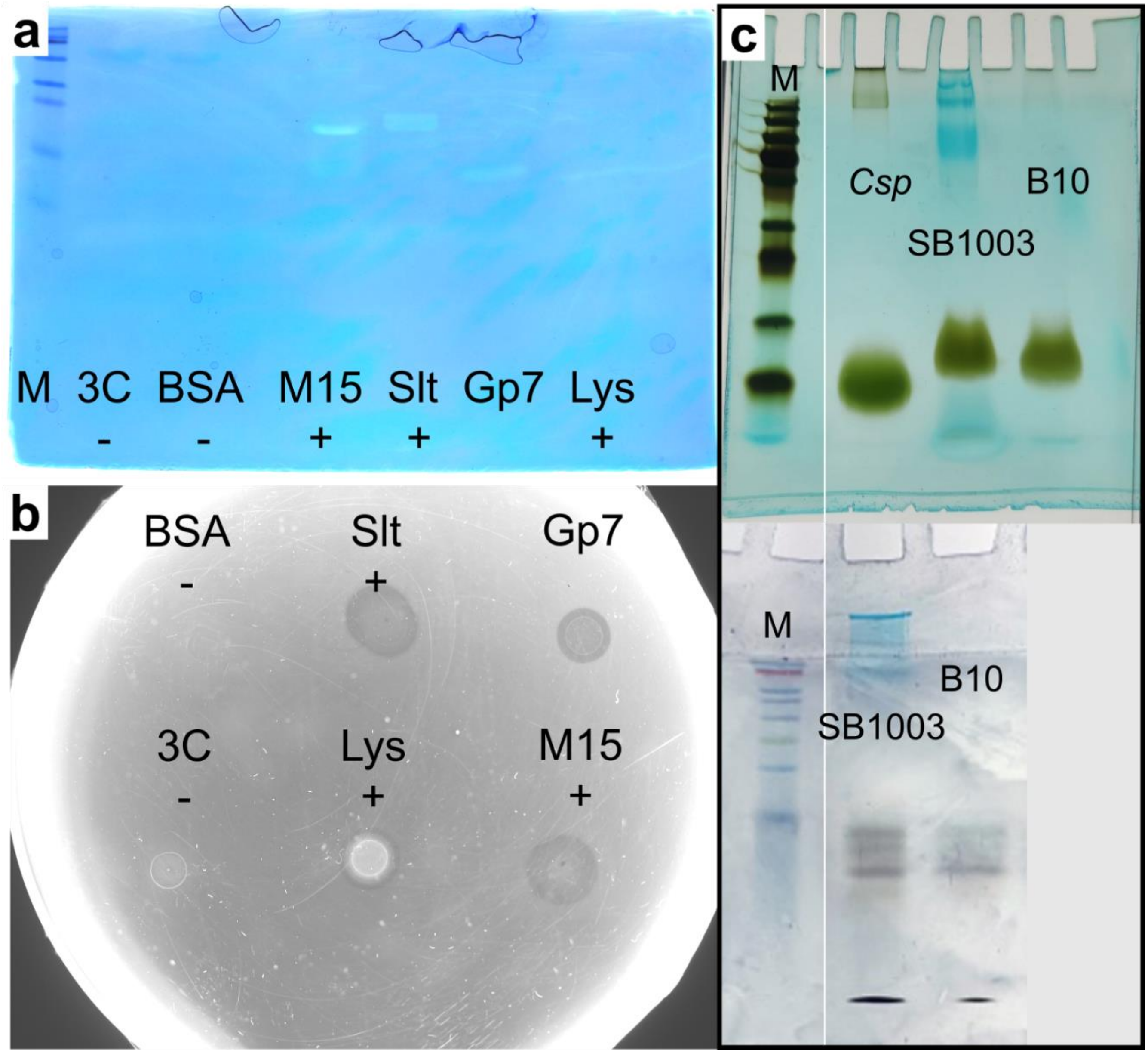

Supp Figure S1: Images of gels and the water agar plate. a-b) Enzymatic assays of Gp7 with zymogram SDS-PAGE gel (a) and water agar plate (b) shown. BSA and 3C protease were used as negative controls, M15 and Slt enzymes of *Rhodobacter* phage Jorvik (1) as well as lysozyme were used as positive controls. c) Gels with LPS samples that were used for Ebor inhibition assay ran on Any kD™ Mini-PROTEAN® TGX™ gradient gel (top) and more diluted samples ran on a 15 % polyacrylamide gel (bottom).

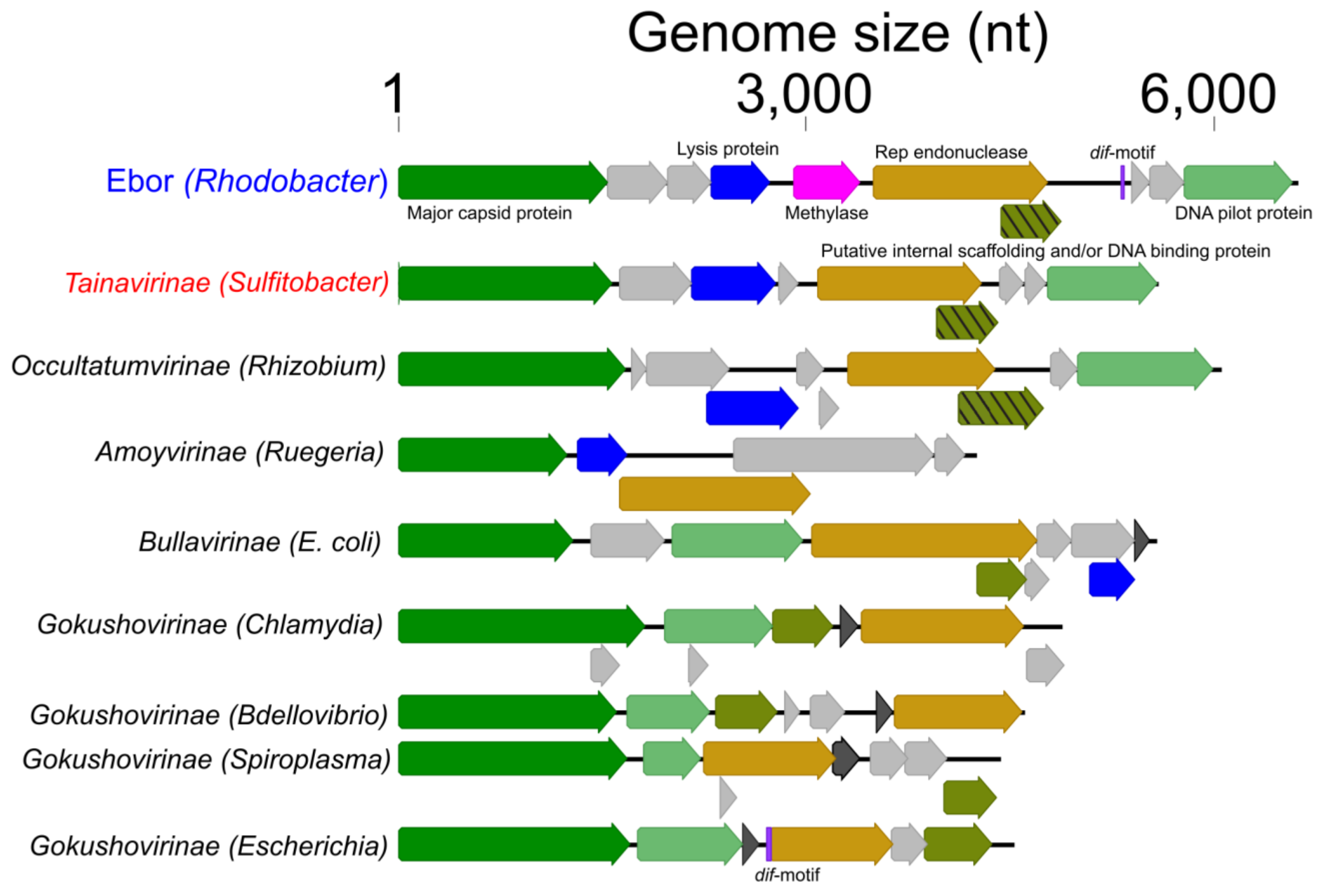

Supp Figure S2: Genome structure of Ebor and select other microviruses. Homologous/analogous proteins are shown in the same colour.

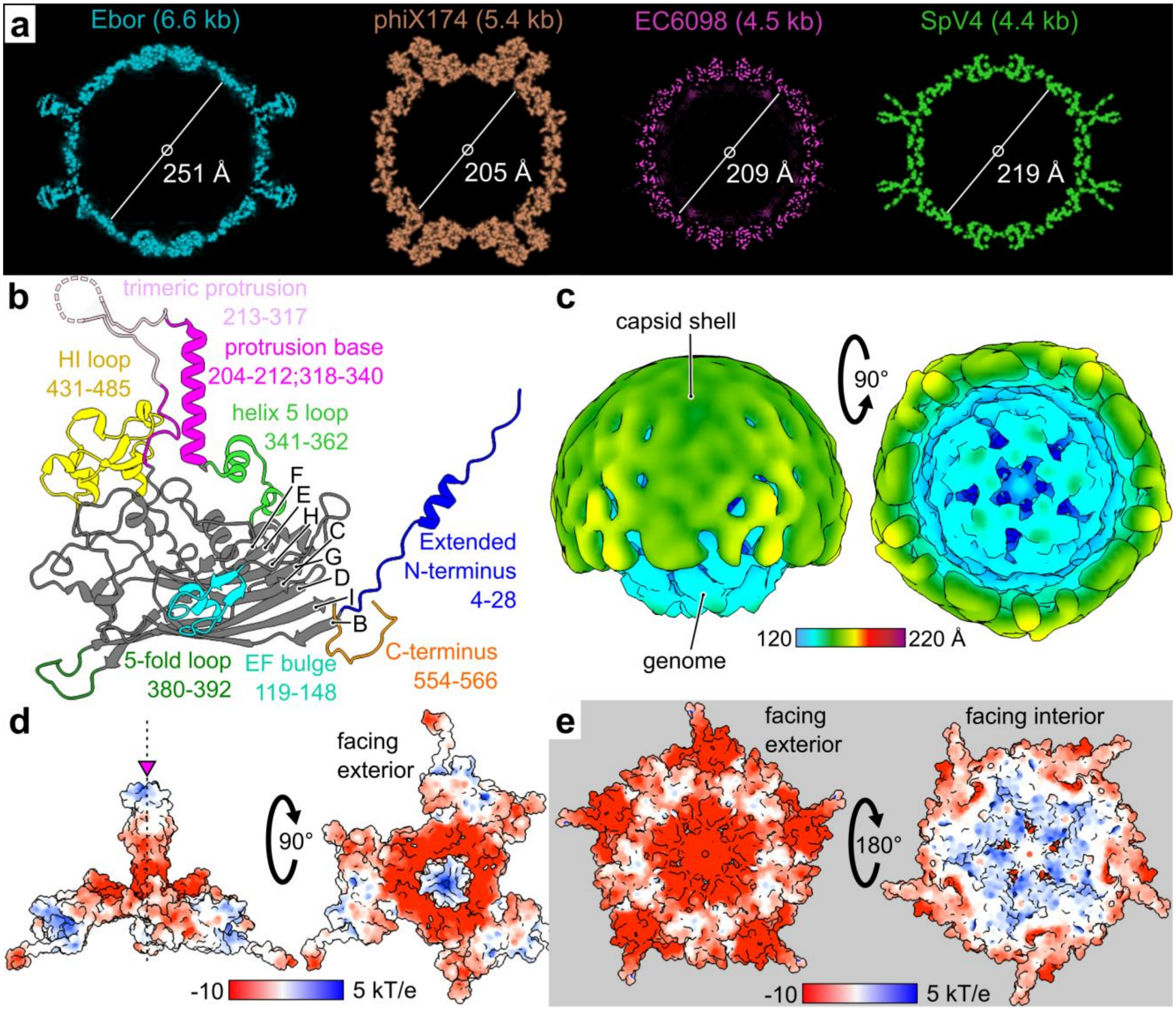

Supp Figure S3: Additional structural analysis of the native virion of Ebor. a) Diameters of individual *Microviridae* phages. Central Z slices of the capsids are shown, the densities of phiX174 and SpV4 were generated by ChimeraX molmap command (2) of PDB bioassemblies 2BPA_A and 1KVP_A respectively. For EC6098, a segmentation of EMD-27397 was used, masking out the central virion density for clarity. b) Ribbon diagrams of the major capsid protein of phage EC6098, PDB code 8DES_A. Regions of interest are highlighted in colour and delimiting residues numbers are shown. c) Map of Ebor virion releasing the genome *in vitro* reconstructed by single particle analysis of variant S120 virions purified using CsCl gradient. d-e) electrostatic potential of Ebor protrusion (d) and penton (e) estimated according to Adaptive Poisson-Boltzmann Solver (3). The AlphaFold2-predicted (4) protrusion loops were connected into a single model with respective subunits of major capsid protein in Coot (5) with the axis of symmetry highlighted (magenta triangle). The colour coding corresponds to the electrostatic potential values of the protein surface calculated at T=298.15K and pH=7.0.

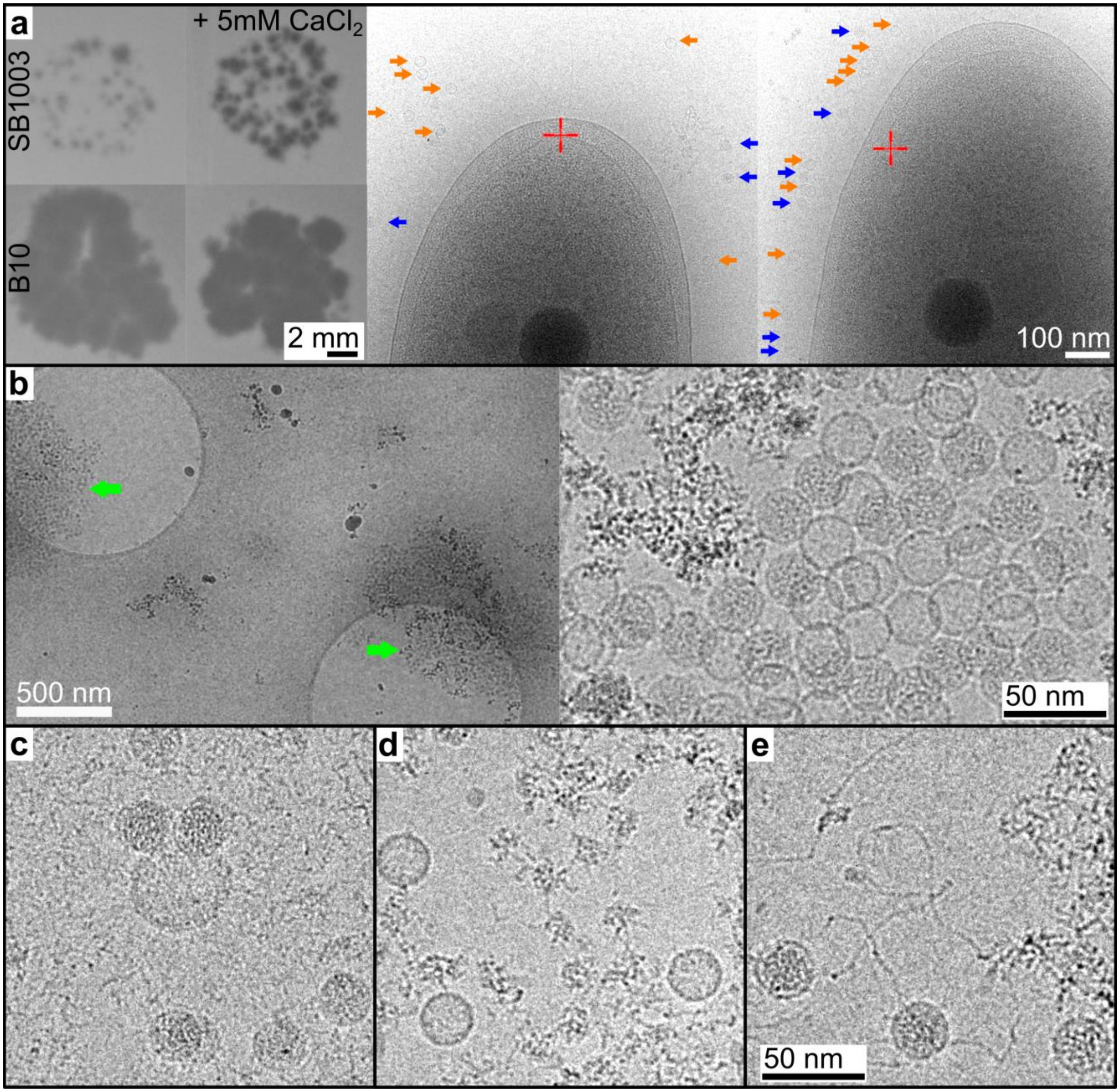

Supp Figure S4: Additional cryo-EM images of Ebor. a) The same virus stock as used in the experiments shown in the **Figure 3d-f)** was imaged after 5 min of incubation with the host strain SB1003 to which 5 mM $CaCl_2$ was added. The effect of the calcium addition on the plaque size is shown in left. b-e) Images of Ebor particles purified using different methods. Ebor variant R120 purified using ion exchange chromatography (b). The particles formed aggregates (green arrows) and the ratio of empty and native particles was close to 1:1. Ebor variant R120 purified using CsCl ultracentrifugation (c), all observed particles were native. Ebor variant S120 purified using sucrose ultracentrifugation (d), all observed particles were empty, the contaminants are likely ejected genome molecules. Ebor variant S120 purified using CsCl ultracentrifugation (e), the particles appear to be releasing their genome. Images were collected at 200 kV using a Glacios TEM equipped with Falcon 4 camera, with the total exposure dose of ~3 $e^{-}/Å^{2}$ in case of cells and 50 $e^{-}/Å^{2}$ in case of purified viruses.

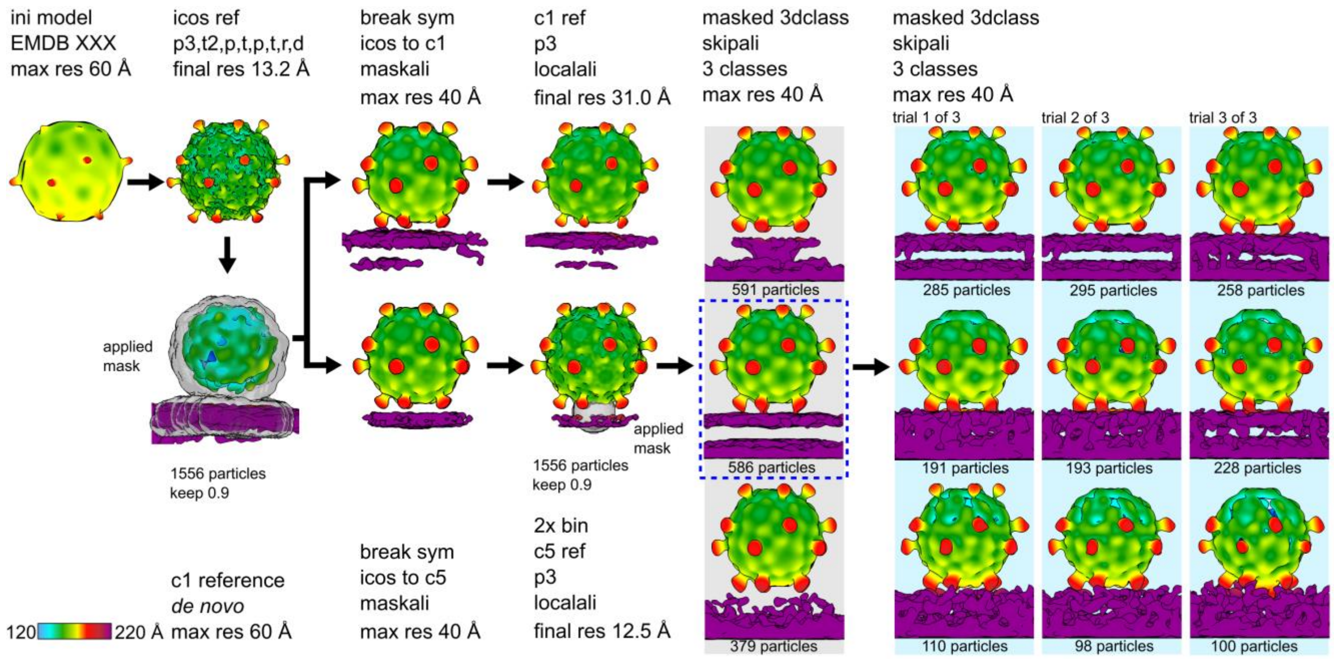

Supp Figure S5: EMAN2 pipeline (6) applied for the subtomogram reconstruction of the dataset of phage Ebor attached to cells. The colour bar indicates the distance from the centre of the capsid. Different iterations of the refinement algorithm are labelled according EMAN2 conventions: p, 3D particle orientation; t, 2D subtilt translation; r, subtilt translation and rotation; d, subtilt defocus refinement.

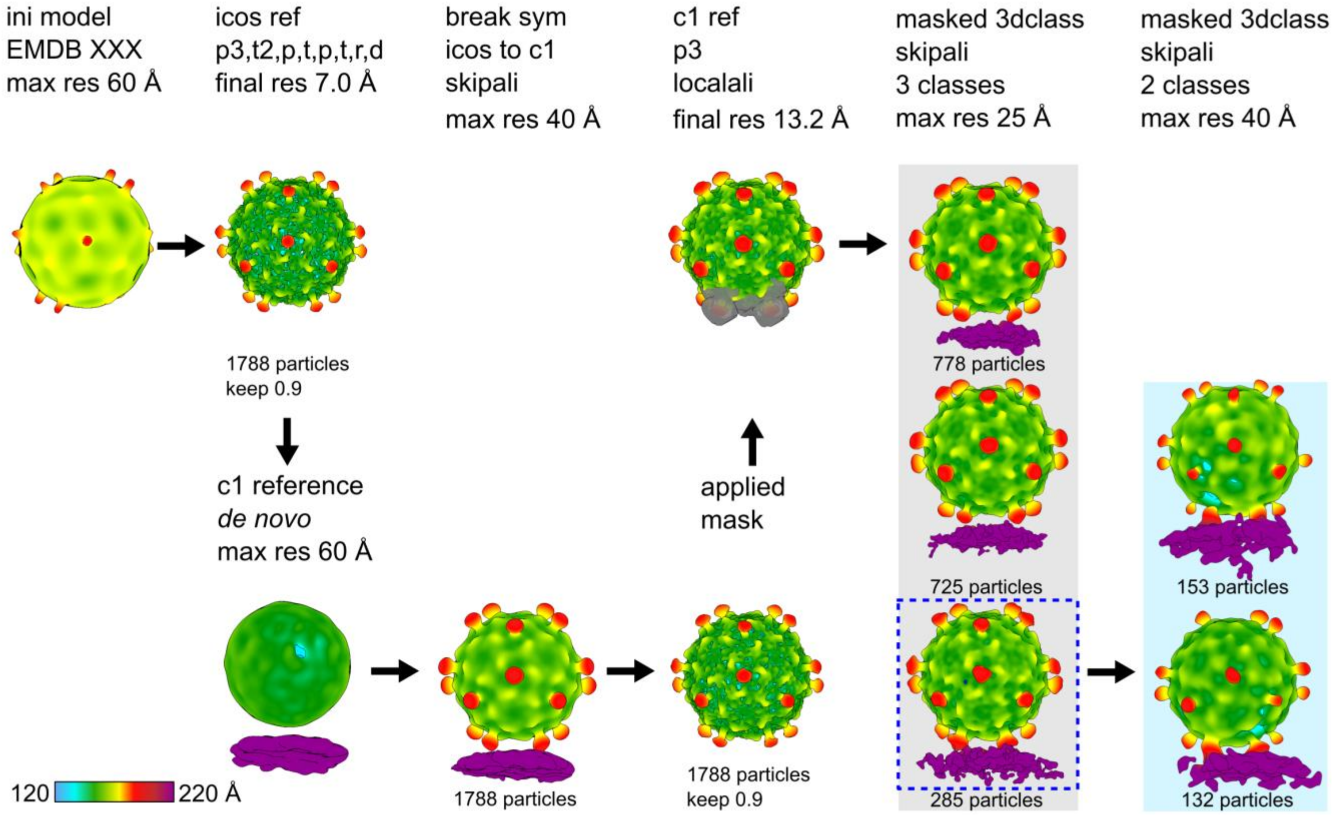

Supp Figure S6: EMAN2 pipeline (6) applied for the subtomogram reconstruction of the dataset of phage Ebor attached to OMVs. The colour bar indicates the distance from the centre of the capsid. Different iterations of the refinement algorithm are labelled according EMAN2 conventions: p, 3D particle orientation; t, 2D subtilt translation; r, subtilt translation and rotation; d, subtilt defocus refinement.

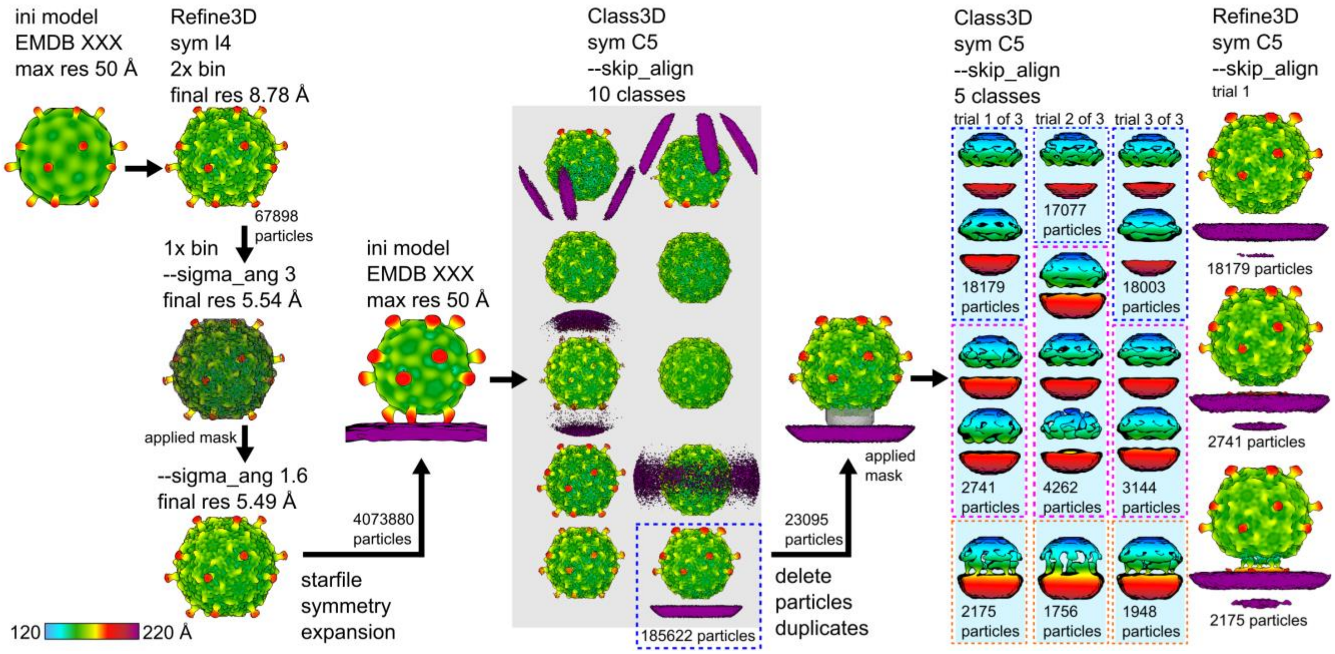

Supp Figure S7: RELION pipeline (7–9) **applied for the single particle reconstruction of the dataset of phage Ebor attached to cells.** The colour bar indicates the distance from the centre of the capsid.

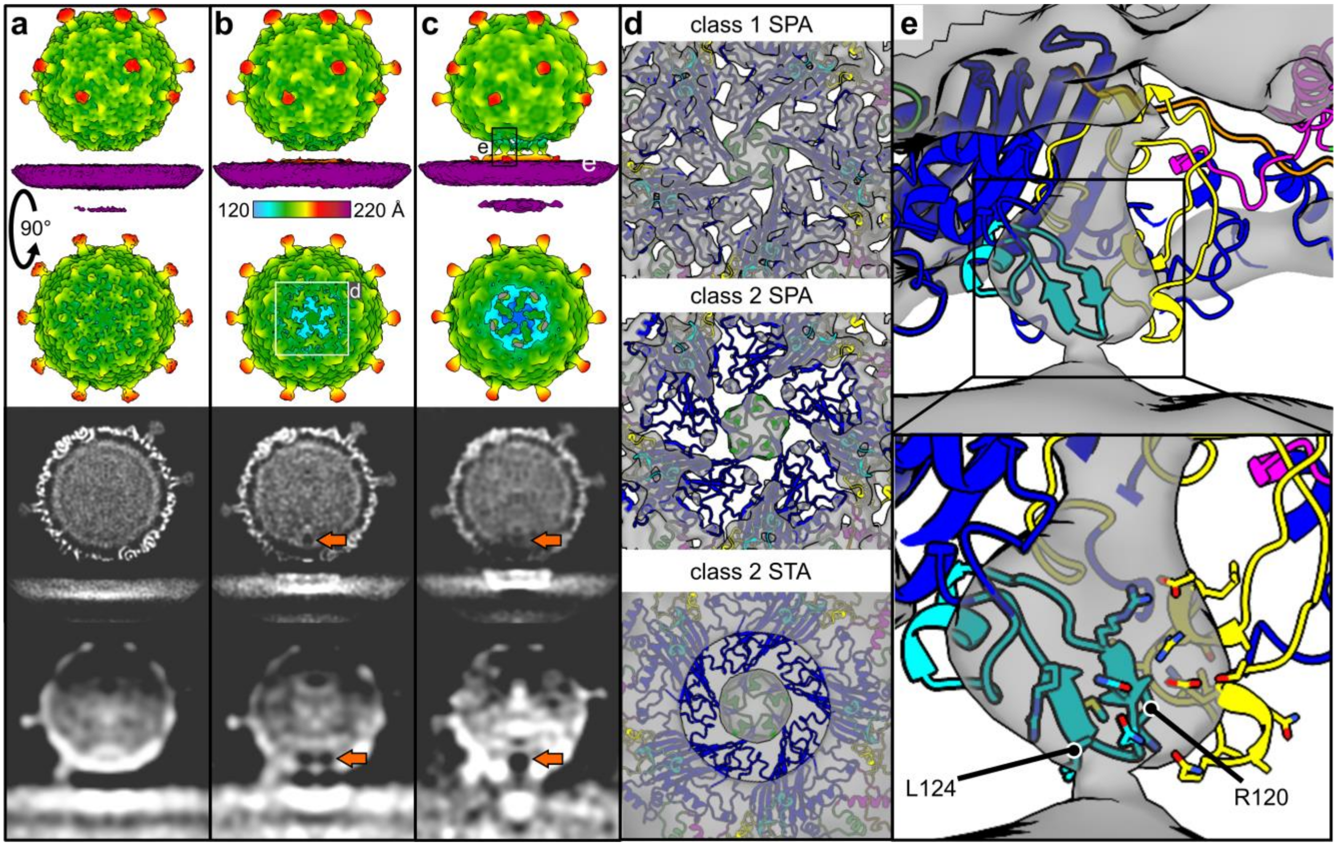

Supp Figure S8: Single particle analysis of Ebor attached to cells. a-c) C5 symmetry maps of the attached particles classified into class 1 (a), 2 (b) and 3 (c) reconstructed by single particle analysis (SPA). Side view, top view and central slice through y axis are shown from top to bottom, with a central slice through y axis of unfiltered maps reconstructed by subtomogram averaging (STA) shown on the very bottom for comparison. The colour bar indicates the distance from the centre of the capsid. The arrows point at the density of an internal cavity formed above the interacting penton. d) Fitting of the major capsid protein model shown as ribbon diagrams into the interacting penton of classes 1 and 2 reconstructed by SPA s and class 2 reconstructed by STA. A similar pore was identified in class 2 reconstructed by both methods, with detached density present right on the 5-fold axis. e) Fitting of the major capsid protein in class 3 map reconstrcuted from SPA shows the density connecting the capsid with the membrane corresponds to the position of EF bulge. Close up of the region is shown in the bottom rectangle. Individual residues are shown as sticks with R120 and L124 highlighted.

Supp Table S1: Cryo-EM and ET data collection and processing information.

Parameters	Single particle analysis				Subtomogram averaging	
	native particle	empty particle	genome-releasing particle	particles attached to cells	particles attached to cells	particles attached to OMVs
**Data collection**						
Magnification	x120000	x120000	x120000	x42000	x45000	x45000
Pixel size [Å]	1.20	1.20	1.20	2.143	2.50	3.15
Camera (energy filter) [manufacturer]	Falcon 4 [TFS]	Falcon 4 [TFS]	Falcon 4 [TFS]	K3 (GIF) [Gatan]	Falcon 4i (Selectris) [TFS]	Falcon 4 [TFS]
Instrument*	Glacios, York	Glacios, York	Glacios, York	Krios II, eBIC	Glacios 2, TFS facility, Eindhoven	Glacios, York
Voltage [kV]	200	200	200	300	200	200
Data collection software	EPU (holey carbon set up)	EPU (holey carbon set up)	EPU (holey carbon set up)	EPU (lacey carbon set up)	Tomo5	Tomo5
Total exposure dose [$e^{-}/Å^2$]	50	50	50	27.878	117.66	115.9
Exposure dose per tilt [$e^{-}/Å^2$]	50	50	50	27.878	3.18	1.9
Number of tilts	1	1	1	1	37	61
Tilt span [°]	0	0	0	0	±54	±60
Tilt increment [°]	NA	NA	NA	NA	3	2
Number of acquired micrographs/tilt series	996	1099	2810	2750	42	71
Number of used micrographs/tilt series	954	764	2095	2742	27	25
Defocus range [µm]	0.5 - 2.5	0.5 - 2.5	0.5 - 2.5	0.75 - 3.0	1.5 - 4.0	1.0 - 3.0
**Data processing**						
Reconstruciton software	RELION3	RELION3	RELION3	RELION5	EMAN2	EMAN2
Symmetry	i4	i4	C5	i4, symmetry expansion, c5	icos, symmetry break to c1 and c5	icos, symmetry break to c1
Initial number of particles	5271	3976	6302	75291	1593	1788
Final number of particles	4935	1984	401	class1=18179, class2=2741, class3=2175	c1=1556, class1=295, class2=193, class3=198	c1=1788, weak1=725, weak2=778, strong1=153, strong2=132
Initial model	emd-50356	*de novo*	*de novo*	emd-50361	emd-50357, break to c1 *de novo*	emd-50357, break to c1 *de novo*
Map resolution [Å]	3.2	3.3	24	7.8, 10.4, 13.4	c1=31, far=40†, close=40†, fused=40†	c1=13.2, weak1=25†, weak2=25†, strong1=40†, strong2=40†
FSC threshold	0.143	0.143	0.143	0.143	0.2	0.2
**Database entry**						
EMDB	50357	50356	50358	50359	50361	50360
PDB	9FFH	9FFG	NA	NA	NA	NA

**all manufactured by TFS; †low passed to this resolution; OMV, outer membrane vesicle; TFS, Thermo Fisher Scientific*

Supp Table S2: Raw data related to the Ebor inhibition assay.

Sample	OMVs	LPS *C.sph.*	LPS B10Δ*gtaI*	LPS SB1003Δ*gtaI*
biol. rep. 1 buffer [PFUs]	2.00E+10	3.20E+08	3.20E+07	6.00E+07
biol. rep. 1 sample [PFUs]	5.00E+08	3.00E+08	1.50E+06	1.10E+07
biol. rep. 2 buffer [PFUs]	2.00E+10	3.20E+08	6.00E+07	6.00E+07
biol. rep. 2 sample [PFUs]	5.30E+08	2.30E+08	1.70E+07	1.20E+07
biol. rep. 3 buffer [PFUs]	2.50E+09	3.20E+08	6.00E+07	6.00E+07
biol. rep. 3 sample [PFUs]	3.00E+07	3.50E+08	1.80E+07	8.00E+06
biol. rep. 1 RI [%]	2.50%	93.75%	4.69%	18.33%
biol. rep. 2 RI [%]	2.65%	71.88%	28.33%	20.00%
biol. rep. 3 RI [%]	1.20%	109.38%	30.00%	13.33%
**average RI [%]**	**2.12%**	**91.67%**	**21.01%**	**17.22%**

OMV, outer membrane vesicle; LPS, lipopolysaccharide; biol. rep., biological replicate; PFU, plaque-forming unit; RI, relative infection

Supp Table S3: Bacterial strains used in this study.

Bacterial species	strain	Purpose	Reference/source
Rhodobacter capsulatus	DE442	source of Ebor	(10)
	SB1003	propagation of Ebor R120, plating strain for inhibition assay	(11)
	SB1003Δ*gtaI*	capsuleless strain, LPS extraction	(12)
	B10	propagation of Ebor S120, host for cryo-EM experiments	(1)
	B10Δ*gtaI*	capsuleless strain, LPS extraction	(13)
Escherichia coli	Stellar	cloning of gp7	TakaraBio
	BL21(DE3)	expression of gp7	Novagen

Supp Table S4: Model refinement statistics. The values were calculated using wwPDB EM Validation server and Molprobity server (14).

**Parameters**		**Model**	
		9FFH	9FFG
**Map**	EMDB code	50357	50356
	Resolution [Å]	3.2	3.3
**Model composition**	Non-hydrogen atoms	3473	3379
	Protein residues	451	437
**R.M.S. deviations**	Bond lengths [Å]	0.26	0.24
	Bond angles [°]	0.55	0.50
**Ramachandran plot**	Favoured [%]	96.42	96.07
	Outlier [%]	0.45	0.00
	Z-score	-1.06 ± 0.39	0.08 ± 0.42
**Validation**	Rotamer outliers [%]	1.83	1.07
	Clashscore	5.05	3.11
	Molprobity score	1.70	1.39
	CaBLAM outliers [%]	3.20	3.00
**Atom inclusion***		0.85	0.84

**at the recommended contour level = 0.1*

Supplementary References

1. Bárdy P, MacDonald CIW, Pantůček R, Antson AA, Fogg PCM. Jorvik: A membrane-containing phage that will likely found a new family within Vinavirales. iScience. 2023 Nov 17;26(11):108104.

2. Pettersen EF, Goddard TD, Huang CC, Meng EC, Couch GS, Croll TI, et al. UCSF ChimeraX: Structure visualization for researchers, educators, and developers. Protein Sci Publ Protein Soc. 2021 Jan;30(1):70–82.

3. Jurrus E, Engel D, Star K, Monson K, Brandi J, Felberg LE, et al. Improvements to the APBS biomolecular solvation software suite. Protein Sci Publ Protein Soc. 2018 Jan;27(1):112–28.

4. Jumper J, Evans R, Pritzel A, Green T, Figurnov M, Ronneberger O, et al. Highly accurate protein structure prediction with AlphaFold. Nature. 2021 Aug;596(7873):583–9.

5. Emsley P, Lohkamp B, Scott WG, Cowtan K. Features and development of Coot. Acta Crystallogr D Biol Crystallogr. 2010 Apr;66(Pt 4):486–501.

6. Chen M, Bell JM, Shi X, Sun SY, Wang Z, Ludtke SJ. A complete data processing workflow for cryo-ET and subtomogram averaging. Nat Methods. 2019 Nov;16(11):1161–8.

7. Zivanov J, Nakane T, Forsberg BO, Kimanius D, Hagen WJ, Lindahl E, et al. New tools for automated high-resolution cryo-EM structure determination in RELION-3. Egelman EH, Kuriyan J, editors. eLife. 2018 Nov 9;7:e42166.

8. Kimanius D, Dong L, Sharov G, Nakane T, Scheres SHW. New tools for automated cryo-EM single-particle analysis in RELION-4.0. Biochem J. 2021 Dec 22;478(24):4169–85.

9. Schwab J, Kimanius D, Burt A, Dendooven T, Scheres SHW. DynaMight: estimating molecular motions with improved reconstruction from cryo-EM images [Internet]. bioRxiv; 2023 [cited 2024 Apr 22]. p. 2023.10.18.562877. Available from: https://www.biorxiv.org/content/10.1101/2023.10.18.562877v1

10. Ding H, Moksa MM, Hirst M, Beatty JT. Draft Genome Sequences of Six Rhodobacter capsulatus Strains, YW1, YW2, B6, Y262, R121, and DE442. Genome Announc. 2014 Feb 13;2(1):e00050-14.

11. Strnad H, Lapidus A, Paces J, Ulbrich P, Vlcek C, Paces V, et al. Complete Genome Sequence of the Photosynthetic Purple Nonsulfur Bacterium Rhodobacter capsulatus SB 1003. J Bacteriol. 2010 Jul;192(13):3545–6.

12. Alim NTB, Koppenhöfer S, Lang AS, Beatty JT. Extracellular Polysaccharide Receptor and Receptor-Binding Proteins of the Rhodobacter capsulatus Bacteriophage-like Gene Transfer Agent RcGTA. Genes. 2023 May 22;14(5):1124.

13. Leung MM, Brimacombe CA, Spiegelman GB, Beatty JT. The GtaR protein negatively regulates transcription of the gtaRI operon and modulates gene transfer agent (RcGTA) expression in Rhodobacter capsulatus. Mol Microbiol. 2012 Feb;83(4):759–74.

14. Chen VB, Arendall WB, Headd JJ, Keedy DA, Immormino RM, Kapral GJ, et al. MolProbity: all-atom structure validation for macromolecular crystallography. Acta Crystallogr D Biol Crystallogr. 2010 Jan 1;66(Pt 1):12–21.
